# Cuprizone feeding induces swollen astrocyte endfeet

**DOI:** 10.1007/s00424-022-02759-8

**Published:** 2022-10-15

**Authors:** Petra Fallier-Becker, Irina Bonzheim, Friederike Pfeiffer

**Affiliations:** 1 Institute of Pathology and Neuropathology, University Hospital Tübingen, Tübingen, Germany; 2 Department of Neurophysiology, Institute of Physiology, Eberhard Karls University of Tübingen, Tübingen, Germany

**Keywords:** Multiple sclerosis, Cuprizone, Astrocytic endfeet, Aquaporin-4, Electron microscopy, Edema, Orthogonal arrays of particles (OAPs)

## Abstract

The cuprizone model is a widely used model to study the pathogenesis of multiple sclerosis (MS). Due to the selective loss of mature oligodendrocytes and myelin, it is mainly being used to study demyelination and the mechanisms of remyelination, as well as the efficiency of compounds or therapeutics aiming at remyelination. Although early investigations using high dosages of cuprizone reported the occurrence of hydrocephalus, it has long been assumed that cuprizone feeding at lower dosages does not induce changes at the blood–brain barrier (BBB). Here, by analyzing BBB ultrastructure with high-resolution electron microscopy, we report changes at astrocytic endfeet surrounding vessels in the brain parenchyma. Particularly, edema formation around blood vessels and swollen astrocytic endfeet already occurred after feeding low dosages of cuprizone. These findings indicate changes in BBB function that will have an impact on the milieu of the central nervous system (CNS) in the cuprizone model and need to be considered when studying the mechanisms of de- and remyelination.

## Introduction

Multiple sclerosis (MS) is a chronic inflammatory disease of the central nervous system (CNS), accompanied by the formation of inflammatory lesions, demyelination, neurodegeneration, glial scar formation, and impaired blood–brain barrier (BBB) function [1]. Changes in astrocytes associated with BBB impairment at early stages of the disease have been described in MS [2].

The cuprizone model is widely used as a model for MS in order to study the mechanisms of de- and remyelination [3]. Systemic administration of the copper chelator leads to the selective loss of oligodendrocytes, which seem to be especially vulnerable to this toxin [4–6]. In contrast to experimental autoimmune encephalomyelitis (EAE), mainly used to study immune cell contribution to the demyelinating disease MS, where the BBB often is disrupted by lymphocyte migration from the bloodstream into the brain parenchyma [7], it is commonly assumed that the BBB is not affected in the cuprizone model [8, 9]. Early investigations in the superior cerebellar peduncles and brainstem of cuprizone-fed mice showed no signs of changes in BBB permeability [10, 11]. Others observed edematous vacuolation in gray and white matter and the occurrence of reactive astrocytes upon cuprizone administration [4, 12] and even hydrocephalus formation in mice when cuprizone was fed at high doses that are not applied anymore [13].

Newer studies using different approaches and more sensitive dyes revisited the question whether the BBB is affected in the cuprizone model. When analyzing the presence of dietary cholesterol in the brain, Berghoff et al. found extravasation of Evans blue dye into the CNS parenchyma, which is not the case without cuprizone administration, although to a lower extent as compared to the situation in EAE [14]. In a follow-up study, the authors could show that hyperpermeability of the BBB and edema formation in the cuprizone model occur even before the onset of demyelination, presumably through the secretion of inflammatory mediators by astrocytes [15]. Interestingly, aquaporin-4 (AQP4) mRNA was upregulated and the diffuse expression of the AQP4 protein occurred together with an increase in brain water content [15]. More recently, loss of polarized AQP4 staining around small brain vessels and diffuse AQP4 immunoreactivity in the brain parenchyma was found after cuprizone treatment [16]. Thus, it is likely that brain water homeostasis and AQP4 distribution are affected in the toxin-induced model of demyelination.

In brain capillaries, endothelial cells constitute the lumen-associated inner layer, comprising the tight physical barrier with their tight junctions. The abluminal side of the endothelial vessel lining is facing a membrane-associated lamina consisting of extracellular matrix. Pericytes are frequently embedded in the basal lamina and astrocytes embracing the vessels by their endfeet [17] contact the basal lamina from the parenchymal side [18]. The membrane of the endfoot is characterized by the presence of orthogonal arrays of particles (OAPs) that consist of the water channel protein aquaporin-4 (AQP4) [19], which is thought to play an important role in cerebral fluid homeostasis and regulation of brain water content [20]. The polar distribution of AQP4 on the surface of the astrocyte is an important prerequisite for proper BBB function [21]. The balance in the expression of two isoforms of AQP4, M1 and M23, has been found to be crucial for proper OAP arrangement and function [22, 23]. Thereby, M23 is normally expressed threefold more abundant [24, 25].

In this study, we tested the hypothesis that BBB impairment and changes in brain water content that have been observed in the cuprizone model are related to changes in astrocyte endfeet and AQP4 isoform expression. Using electron microscopy, we show the presence of swollen astrocytic endfeet and edema after cuprizone feeding. In addition, we detected rearrangement of OAPs and changes in RNA expression of AQP4 isoforms, indicating a loss of polarity and subsequent BBB impairment induced by cuprizone feeding.

## Methods

### Animals

Ten-week-old male and female mice were fed with either pellets containing 0.2% cuprizone (ssniff Spezialdiäten GmbH, Soest, Germany) or with standard rodent chow as described before [26]. Corpus callosum and neocortex were analyzed at the following time points: 5 weeks of cuprizone feeding (9 mice), 5 weeks of cuprizone feeding plus 1 week of recovery with normal food (5 mice), 5 weeks of cuprizone feeding plus 5 weeks of recovery with normal food (7 mice), or controls that always received normal food (8 mice).

All experiments were performed in accordance with the German Animal Welfare Law and the allowance of the (local) authorities (Regierungspräsidium Tübingen).

### Transmission electron microscopy

For electron microscopy, mice were anesthetized as with a mixture of ketamine (120 mg/kg) and xylazine (8 mg/kg) and transcardially perfused with a mixture of 4% PFA/4% glutaraldehyde (GA) in PBS. Corpus callosum and neocortex were dissected and post-fixed in the same fixative for 4 h. After washes in 0.1 M cacodylate buffer (pH 7.4), samples were additionally fixed in 1% OsO_4_, dehydrated in an ethanol series (50, 70, 96, and 100%) during which 70% ethanol was saturated with uranyl acetate for contrast enhancement. After the dehydration was completed by incubation in propylene oxide, specimens were embedded in Araldite (Serva, Heidelberg, Germany) which was hardened at 60 °C for 48 h. Semithin Sects. (400 nm) and ultrathin Sects. (60 nm) were cut on an FCR Reichert Ultracut ultramicrotome (Leica, Bensheim, Germany). The semi-thin sections were dried on glass slides, and sections were stained for 1 min with Richardson’s solution at 70 °C and rinsed with distilled water. From the selected region, ultra-thin sections were made, mounted on Pioloform-coated copper grids, and contrasted with lead citrate and saturated uranyl acetate. Images were acquired with an EM10A electron microscope (Carl Zeiss, Oberkochen, Germany) and a digital camera (Tröndle, Germany). Image plates were generated with Adobe Photoshop and annotations were added. No image modifications were applied. For statistical analysis, GraphPad Prism 9 was used.

### Freeze fracture

For freeze-fracture analysis, parts of the neocortex from a mouse fed with 0.2% cuprizone for 5 weeks with a subsequent 5-week recovery phase and the respective control littermate were fixed with 2.5% glutaraldehyde in 0.1 M cacodylate buffer at 4 °C overnight. Specimens were cryoprotected and further handled as previously described [27]. Image plates were generated with Adobe Photoshop. No image modifications were applied.

### Quantitative real-time PCR

Mice were anesthetized with a mixture of ketamine (120 mg/kg) and xylazine (8 mg/kg) before transcardial perfusion with 4% paraformaldehyde (PFA) in 0.01 M phosphate-buffered saline (PBS). Brains were dissected and coronally cut before they were fixed in 4.5% formaldehyde overnight and embedded in paraffin as described before [28] or directly embedded into TissueTek (OCT compound; Sakura) and frozen and stored at − 80 °C [26]. Sections were generated with a Microm HM355S (Thermo Fisher Scientific, Waltham, MA, USA) or a Leica CM1950 cryotome (Leica, Wetzlar, Germany).

RNA of tissue from cuprizone-fed and control mice was extracted from macrodissected 5-µm paraffin sections (experiment 1) or 16-µm-thick cryosections that have been embedded in Tissue Tek (experiment 2) using the Maxwell RSC RNA FFPE Kit and the Maxwell RSC Instrument (Promega, Madison, WI, USA) according to the manufacturer’s instructions. For FFPE material, sections were dewaxed in xylene and ethanol for 5 min two times, respectively. Tissue was microdissected in Maxwell incubation buffer and RNA was extracted according to the Maxell RSC RNA FFPE Kit manual. RNA was transcribed into cDNA using Superscript II reverse transcriptase (Thermo Fisher Scientific) and a mix of Oligo(dT) primer (Promega, Madison, WI, USA) and random hexamers (Thermo Fisher Scientific). qRT-PCRs were carried out in duplicates with the QuantiTect SYBR Green PCR Kit (Qiagen, Hilden, Germany) using primers and cycling conditions as previously described [22] and a LightCycler 480 System for detection (Roche Applied Science). Data were analyzed using the 2^−ΔΔCp^ method. Animals and experimental groups are listed in Table 1.

**Table 1:** Changes in aquaporin-4 isoform expression in cuprizone-treated mice

Experimental group	M23/M1 ratio	M23/M1 ratio
	Experiment 1	Experiment 2
Control	3	2.10
Cuprizone 5 weeks	1.96	0.97
Cup 5 weeks + 1-week remyelination	2.03	n.a
Cup 5 weeks + 5-week remyelination	2.36	1.23

Quantitative real-time PCR (qRT-PCR) revealed changes in expression of RNA encoding for the two AQP4 isoforms M1 and M23. After cuprizone feeding and during the recovery phases, the M23/M1 ratio decreased in both experimental sets analyzed

## Results

### Increased edema formation at astrocyte endfeet after cuprizone feeding

**Fig. 1:**
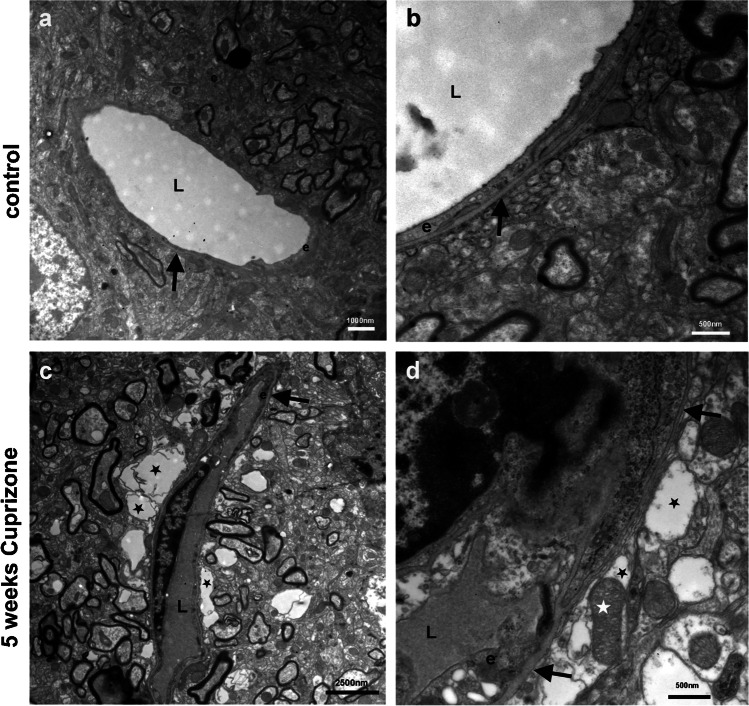

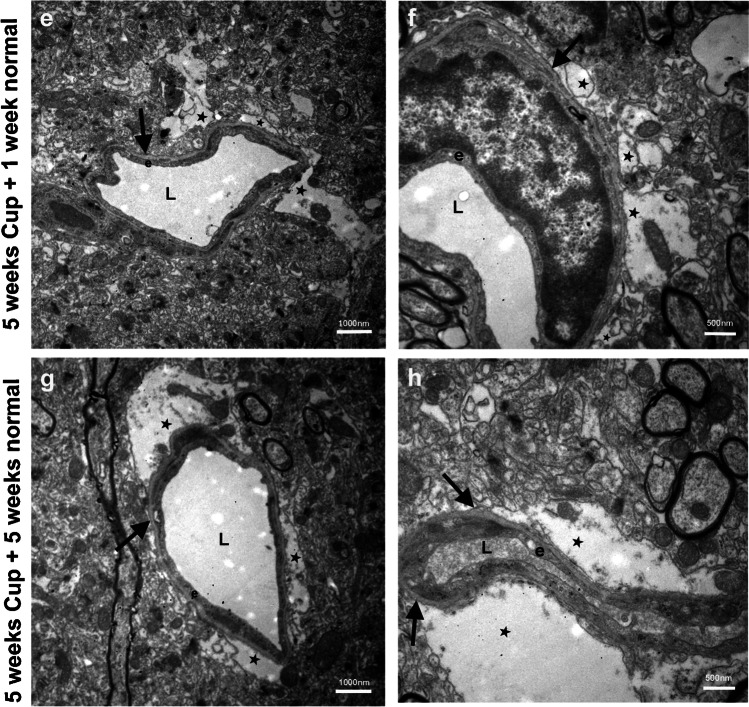
Ultrastructure of blood vessels in the neocortex of cuprizone-treated mice. **a**, **b** Normal-appearing blood vessels in the neocortex of control mice. Note the distinct structure of the basal lamina (black arrows). **a** Low-power image; bar represents 1000 nm. **b** High-power image; bar represents 500 nm. **c**, **d** After 5 weeks of feeding cuprizone pellets, edemas start to appear adjacent to the basal lamina (black arrows). These edemas represent swollen astrocytic endfeet (black asterisks), as they contain organelles, e.g., mitochondria (white asterisk in **d**). **c** Low-power image; bar represents 2500 nm. **d** High-power image; bar represents 500 nm. **e**, **f** After 5 weeks of cuprizone feeding with an additional week of feeding with normal food, large edemas are visible and even increased as compared to (**c**) and (**d**) (black asterisks). Black arrows indicate the basal lamina. **e** Low-power image; bar represents 1000 nm. **f** High-power image; bar represents 500 nm. **g**, **h** After 5 weeks of cuprizone feeding with additional 5 weeks of feeding with normal food, large edemas are visible (asterisks). Black arrows indicate the basal lamina. **g** Low-power image; bar represents 1000 nm. **h** High-power image; bar represents 500 nm. L, vessel lumen; e, endothelial cell

**Fig. 2:**
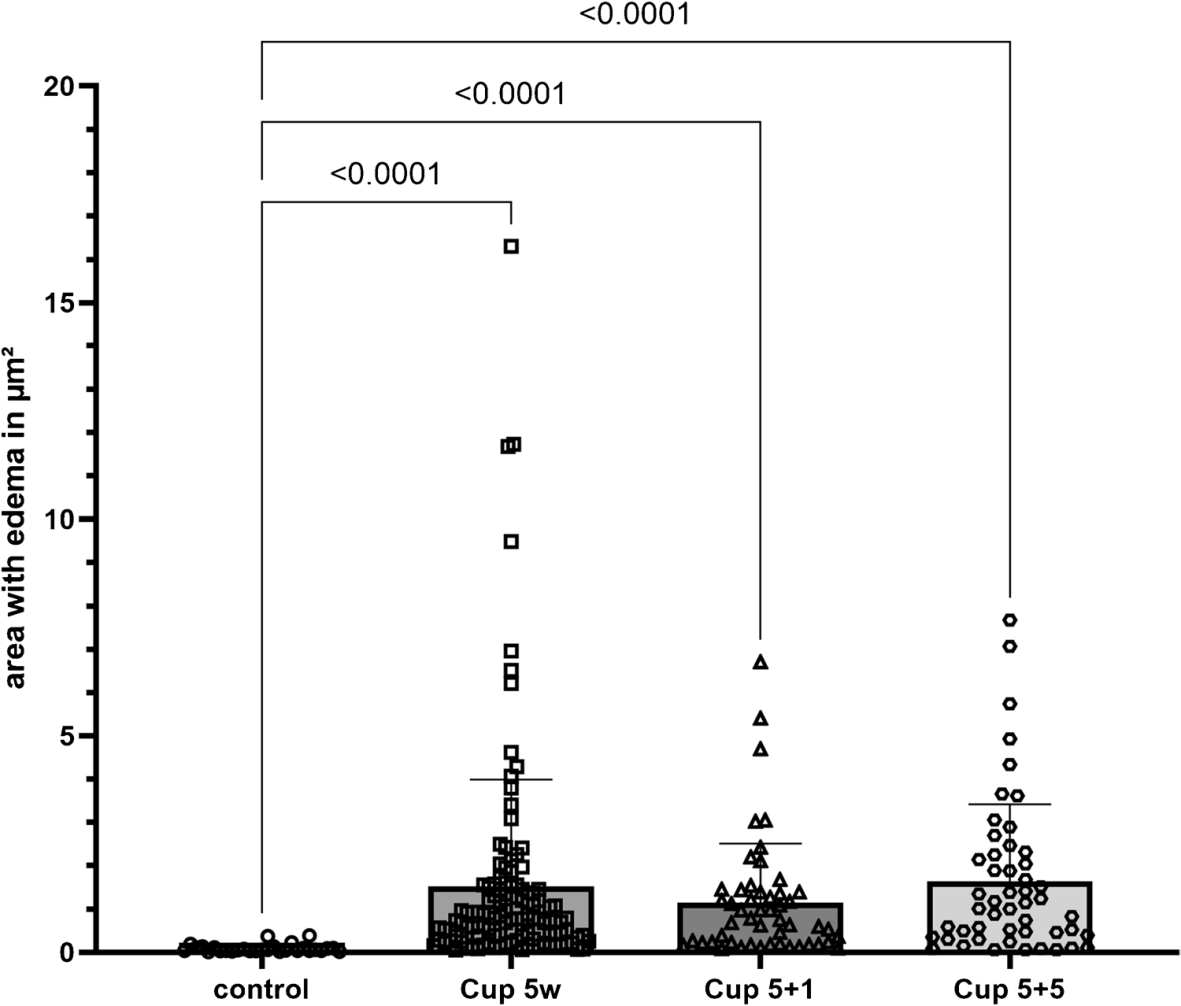
Area with edema in all experimental groups. Quantification (number of occurrence) and size distribution (plotted on *y*-axis) of swellings occurring directly adjacent to the basement membrane and thus localized in astrocytic endfeet in all experimental groups. Ultrastructural images from *n* = 3 mice per group were analyzed and the size of the vacuoles was measured (µm^2^). Each symbol represents one vacuole. The number of symbols represents the frequency, with which vacuoles and edemas were detected on the images taken from each group. The number is lower in the control group, as there was hardly any vacuole detectable next to the basement membranes. Shapiro–Wilk test revealed non-normal distribution of the data. Kruskal–Wallis test was applied with subsequent Dunn’s multiple comparison test. *P* values (as compared to control) are indicated in the graph

To assess morphological changes at the BBB, we performed ultrastructural analysis around blood vessels in the brain parenchyma of control and cuprizone-fed mice, as well as after 1 or 5 weeks of recovery (Fig. 1). In the neocortex of control mice (Fig. 1a, b), we found non-disrupted vascular organization with endothelial cells lining the vessel lumen, a clearly recognizable basal lamina, and continuous coverage of the abluminal vessel wall by astrocytes. There were hardly any vacuoles detectable at the abluminal side of the vessels. After 5 weeks of cuprizone feeding, we observed obvious changes in vessel organization in the neocortex (Fig. 1c, d). Although endothelial cells and the basal lamina appeared to be intact, we frequently saw swollen astrocytic endfeet that appeared as holes around the vessels (Fig. 1c) and characteristically contained organelles such as mitochondria (Fig. 1d). These findings were even more pronounced in the 1 week (Fig. 1e, f) and 5 weeks (Fig. 1g, h) of recovery groups. We frequently found large holes around vessels that sometimes even resembled edemas and the presence of organelles within these structures indicating that they are swollen astrocytic endfeet. Thus, morphological changes that were induced by cuprizone feeding persisted throughout the recovery phase, and edema formation was rather increased than reverted (Fig. 2).

### Changes in the formation of OAPs

**Fig. 3:**
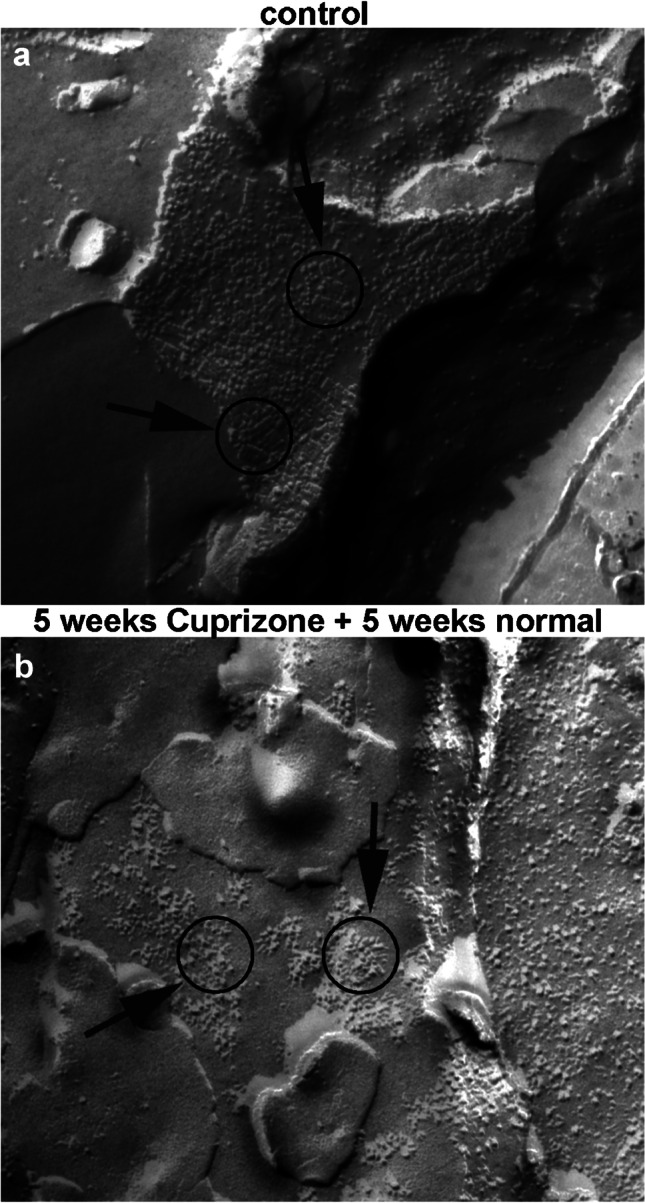
Freeze-fracture analysis of orthogonal arrays of particles. **a** Orthogonal arrays of particles (OAP, circles and arrows) along blood vessels in control mice. **b** After 5 weeks of cuprizone feeding plus additional 5 weeks on normal food, clusters of particles are still present (circles and arrows), but more dispersed and not arranged in arrays anymore, as compared to the control. Scale bars represent 100 nm

The water channel protein AQP4 is the main constituent of OAPs, and its expression is critical for brain water transport [29]. Its isoform composition has been shown to be important for volume regulation in astrocytes [30] and is often altered under pathological conditions [22]. Therefore, we analyzed the distribution of OAPs by applying freeze-fracture technique on the control tissue (Fig. 3a) as well as after 5 weeks of cuprizone feeding with subsequent 5 weeks of recovery (Fig. 3b). In control mice (circles in Fig. 3a), we found evenly arranged OAPs at the endothelial-astrocytic interface, indicating a healthy blood–brain barrier. After 5 weeks of cuprizone feeding with additional 5 weeks of feeding normal food, there were still particles detectable at the endothelial-astrocytic interface, but the organization into OAPs was disturbed (circles in Fig. 3b) and the particles were diffusely distributed along the astrocytic membrane, indicative of disturbed water homeostasis.

In order to verify whether the altered distribution of OAPs was due to changes in isoform expression of AQP4 induced by cuprizone feeding, we performed RT-qPCR on all four experimental groups (control, 5 weeks of cuprizone feeding, 5 weeks of cuprizone feeding with 1 week of recovery, 5 weeks of cuprizone feeding with 5 weeks of recovery) in two independent experiments. The ratio of expression levels between AQP4 isoforms M23 and M1 was subsequently calculated. Control brains showed the highest ratio (2.10 and 3, respectively), which decreased to the lowest ratio in the 5 weeks of cuprizone feeding group (0.97 and 1.97, respectively). The ratio was again increased after 5 weeks of recovery (2.36 and 1.23, respectively), but remained lower as compared to the control animals (Table 1). These data indicate a change in AQP4 isoform expression as a response to cuprizone administration.

## Discussion

We observed ultrastructural changes around blood vessels in the brain parenchyma upon systemic administration of the copper chelator cuprizone in low dosage. In particular, astrocyte endfeet displayed clear swellings around most vessels after cuprizone administration that were not observed in control mice. Concomitantly, OAPs were redistributed at astrocyte endfeet and AQP4 isoform expression was changed.

It has long been assumed that cuprizone feeding does not affect the integrity of the BBB [8, 9] due to the lack of apparent leakiness and barrier dysfunction. Nevertheless, very early studies using high-dosage cuprizone feeding resulted in some animals forming hydrocephalus [31]. Since then, the dosage of cuprizone administered by food has been reduced and studies focused on its effect on oligodendrocytes and myelin [32], but it is also clear that cuprizone-induced changes vary considerably between CNS regions [33]. Only recently, changes in barrier permeability have been observed [14, 15] in commonly applied protocols that are used to study demyelination.

We used pellets containing 0.2% cuprizone in our experiments. This experimental setup results in incomplete but clearly detectable demyelination accompanied by functional impairment of compound action potentials, which recovered when cuprizone feeding was stopped after 5 weeks, as described earlier [26]. In addition, CC1-positive, mature oligodendrocytes that represented 40% of all cells in the corpus callosum of control mice were reduced to 30% after feeding 0.2% cuprizone pellets for 5 weeks. After switching back to normal food, oligodendrocyte numbers recovered to 40% already after 1 week of recovery phase [26]. These data showed a detectable but incomplete reduction of mature oligodendrocytes and myelin that could be regenerated once the toxin was deprived.

In the current study, we demonstrate ultrastructural changes at the brain vasculature upon administration of pellets containing 0.2% cuprizone, a dose that is considered rather low in comparison to other experimental paradigms that have been used in the literature [32], especially when comparing it to mixing fresh powder into the chow every day at the same dose [34].

Earlier ultrastructural studies on postmortem tissue observed alterations in endothelial cells, pericytes, and astrocytes in brain biopsies from patients suffering from chronic progressive MS, providing evidence for BBB abnormalities even in the absence of active inflammation [35]. Interestingly, swellings of astrocytic endfeet were observed in most patients, with a variable proportion of capillaries showing this pathological change. The results of these studies indicated that BBB function did not return to its normal state in chronic MS [35]. While we have shown earlier that 0.2% cuprizone-induced demyelination was restored and the function of callosal fibers had returned back to almost normal levels after 5 weeks of recovery [26], the astrocytic swellings we describe here did not resolve, even after 5 weeks of recovery, indicating that the damage in this particular cell type is more persistent. Thus, morphological changes at the vasculature, in particular astrocyte endfeet, that were induced by cuprizone feeding persisted throughout the recovery phase, and edema formation was rather increased than reverted. The occurrence of edema formation in the absence of inflammation that has been observed in patients suffering from progressive MS renders the cuprizone model suitable to study these specific changes and the impact these alterations will have on the success of regenerative processes and possible therapies to treat MS.

During EAE, the BBB often is disrupted by lymphocyte migration from the bloodstream into the brain parenchyma [7, 36, 37]. It has been assumed that the BBB is not affected in the cuprizone model [8, 9], as there is no obvious sign of BBB breakdown and the demyelination is not caused by peripheral immune cells [32], although some T-cell recruitment to the CNS eventually happens [38–40]. However, astrogliosis and reactive astrocytes can occur in a regionally specific way in the cuprizone model [41, 42]. Since the toxin is systemically administered, it also affects peripheral organs. Its atrophic effect on the spleen and thymus leads to the suppression of peripheral immune cell function, providing an explanation for the rare appearance of T and B cells in the CNS regardless of the BBB state [43]. Newer studies imply that it also directly causes functional impairment of the BBB [14, 15].

We provide evidence for a direct effect on astrocyte endfeet, where the altered expression of AQP4 subunits in the brain parenchyma of cuprizone-treated mice leads to vacuole formation and edema around brain vessels. These changes persisted even 5 weeks after removal of cuprizone-containing food, indicating that the changes in astrocytes and edema formation occur independently from demyelination.

Antibodies directed against AQP-4 are characteristically found in patients suffering from neuromyelitis optica (NMO), a demyelinating disease of the CNS that mainly affects the optic nerve and the spinal cord [44]. Specific antibodies targeting AQP4 appear early in the disease in the serum of a majority of patients affected with NMO that are destructive to astrocytes, perturbing water homeostasis in the CNS [44–47]. Patients diagnosed with AQP4 antibody-associated demyelinating optic neuritis frequently suffer from neuropathic pain and optic nerve head swelling [48], resembling the edema formation revealed by electron microscopy in our study.

In the cuprizone model, demyelination of the optic nerve seems to be dose and/or time dependent. While feeding 0.2% cuprizone for 12 weeks induced a reduction of myelin basic protein (MBP) in the optic nerve [49], feeding 0.2% cuprizone for 5 weeks did not cause detectable demyelination [50]. Feeding 0.1% cuprizone-containing food for 12 weeks did not change myelination of the visual pathway, but induced changes in the optic nerve proteome [51], in line with the assumption that cellular changes can occur independently of detectable demyelination in this model. The cuprizone-induced changes in APQ4 isoform expression and astrocyte endfoot swellings we observed in our study also occurred independent from the myelination state.

The regulation of brain water content by polarized astrocytes is affected in several diseases and disease models. We have previously shown that astrocyte polarity/AQP4 localization is lost as a response to increased leukocyte trafficking across the BBB in the EAE model, and that this loss of polarity was responsible for edema formation [52]. In human glioblastoma, OAP formation is disturbed in spite of upregulated AQP4 protein [53]. Here, we observe changes in AQP4 isoform expression and the disturbance of OAP organization after feeding low doses of cuprizone as a sign of BBB disturbance that persisted after 5 weeks of recovery. Thereby, freeze fracture is the only method established to date that will reveal the polar distribution of OAPs in astrocytes.

Quantitative RT-PCR showed an upregulation of AQP4 isoform M1 as compared to M23 after cuprizone feeding. It has been shown that under normal conditions in the brain, M23 is expressed about three times higher as compared to M1 [24, 25], and that both isoforms have opposing actions on OAP formation [23]. For proper OAP formation, both isoforms are necessary, M1 and M23. Expressing only M1 in cultured cells results in no OAP formation, while expressing only M23 leads to massive formation of OAP “lattices” [23]. Our results from qRT-PCR of two independent experiments showed that the ratio between M23 and M1 was highest in control brains (values between 2 and 3), representing the situation in healthy brains. After 5 weeks of cuprizone feeding, this ratio was greatly diminished to 1.96 and 0.97, respectively, and did not return to control levels during the 5-week recovery phase. These findings are comparable to the data obtained in mouse astrocytes representing a glioblastoma model as well as in human tissue from glioblastoma, where we found the M23/M1 ratio to be 1.94 [22]. The decrease in the M23/M1 ratio indicates a loss of polarity of the astrocyte and hence an impairment of the BBB that is in line with the increase in astrocyte endfeet swellings and edema formation we observed in this study.

Together, our findings demonstrate that astrocytes react to the intoxication with cuprizone in the absence of lymphocyte trafficking to the CNS and before apparent BBB breakdown manifests. Together with a significant increase in the number swollen endfeet and edema at the brain vasculature, we observed a change in the expression pattern of OAPs, comparable to hypoxia cluster in glioblastomas with a low M23/M1 ratio [22]. All of these astrocytic changes were not reverted within the 5-week recovery phase that was monitored.

What is the underlying mechanism of astrocytes upregulating and redistributing water channel proteins? One can hypothesize that astrocytes upregulate AQP4 as a reaction to contact with the toxin, and in an attempt to wash out the toxic compound, but this clearly needs further investigations. It has recently been shown that the lack of AQP4 in the retina during inflammation resulted in impaired clearing of retinal swellings and provoked astrogliosis and loss of ganglion cells [54], indicating a protective role of AQP4 in autoimmune diseases in the CNS.

Our findings are in line with studies suggesting that astrocytes and vascular cells are affected by systemic administration of cuprizone, leading to altered function of the BBB. These findings should be kept in mind when interpreting findings from studies using the cuprizone model and analyzing remyelination.
